# Understanding and Predicting Social Media Use Among Community Health Center Patients: A Cross-Sectional Survey

**DOI:** 10.2196/jmir.3373

**Published:** 2014-11-26

**Authors:** Carl L Hanson, Josh West, Rosemary Thackeray, Michael D Barnes, Jordan Downey

**Affiliations:** ^1^Department of Health ScienceCollege of Life SciencesBrigham Young UniversityProvo, UTUnited States

**Keywords:** social media, community health centers, medically underserved area

## Abstract

**Background:**

The use of social media by health care organizations is growing and provides Web-based tools to connect patients, caregivers, and providers.

**Objective:**

The aim was to determine the use and factors predicting the use of social media for health care–related purposes among medically underserved primary care patients.

**Methods:**

A cross-sectional survey was administered to 444 patients of a federally qualified community health center.

**Results:**

Community health center patients preferred that their providers use email, cell phones for texting, and Facebook and cell phone apps for sharing health information. Significantly more Hispanic than white patients believed their providers should use Facebook (*P*=.001), YouTube (*P*=.01), and Twitter (*P*=.04) for sharing health information. Use and intentions to use social media for health-related purposes were significantly higher for those patients with higher subjective norm scores.

**Conclusions:**

Understanding use and factors predicting use can increase adoption and utilization of social media for health care–related purposes among underserved patients in community health centers.

## Introduction

Social media includes “Web-based and mobile technologies used to turn communication into interactive dialog between organizations, communities, and individuals” [1]. Health care organizations have begun to recognize the value of these technologies for connecting, communicating, and collaborating [2-8] with social media interactions categorized as patient-patient, clinician-patient, public health-consumer, researchers-patient, and corporate-consumer [9]. As they have been applied to health care, new terms such as Medicine 2.0, Health 2.0, and eHealth have emerged to describe the plethora of Web-based tools of the second generation Internet (Web 2.0) used to connect patients, caregivers, and health professionals [10]. Eysenbach has defined Medicine 2.0 as “Web-based services for health care consumers, caregivers, patients, health professionals, and biomedical researchers that use Web 2.0 technologies and/or sematic Web and virtual reality approaches to enable and facilitate specifically (1) social networking, (2) participation, (3) apomediation, (4) openness, and (5) collaboration within and between these user groups” [11].

Several motives explain the application of social media in health care [12]. These motives include information seeking about disease treatment and medicines [12-14], social support between 2 or more people with the same illness [12,15,16], improved efficiency and quality of care [6,17], improved relationships with providers [5], and self-care and self-management [18]. Patients of clinical providers are increasingly likely to go online to find advice and share information about their condition through the Internet and social media platforms than ever before [19]. Fisher and Clayton [20] have assessed patient interest in social media for health care purposes. Their findings revealed that 83% of patients used some form of social media and more than half wanted their providers to use it for health care (ie, share health information updates, communicate, and/or help manage health problems).

In the United States, Federally Qualified Health Centers (FQHCs), such as community health centers, are an important part of the health care system. They provide comprehensive primary and preventive care most often to medically underserved and disadvantaged community members. As the Patient Protection and Affordable Care Act (PPACA) is implemented, FQHCs will be under greater pressure to achieve the “triple aim” of improving affordability, health status, and patient experience [21]. Recent studies have demonstrated the potential value of mobile technologies for promoting access, effective patient-provider communication, and adherence among the underserved [21-23]. Although social media use is promising for health care purposes, effective use among racially and ethnically underserved communities will require an understanding of the adoption and utilization barriers for providers as well as patients [24,25]. For minority patient populations, these barriers may include, but are not limited to, lack of perceived benefit, increased work and time required to use the technology, computer knowledge and skills, access to computers, technology fear/anxiety, lack of cultural relevance, and privacy and trust concerns [24].

Despite a recent call for additional research on social media and health information seeking among the underserved [26], no studies have explored social media use among those served by FQHCs. The purpose of this study was to determine use and factors predicting intentions to use social media for health information and support among medically underserved primary care patients in a community health center. Research questions included:

1. To what extent do patients use social media?
2. What are patient preferences for how health care providers should use social media to communicate?
3. What factors from the theory of planned behavior (TPB) predict intentions use of social media for health care–related purposes?

Although it is critically important to monitor race/ethnicity on health information seeking to reach those in most need [26], this study also compared social media use and theoretical constructs between the 2 largest groups in the sample: white and Hispanic patients.

## Methods

### Theoretical Framework

**Figure 1:**
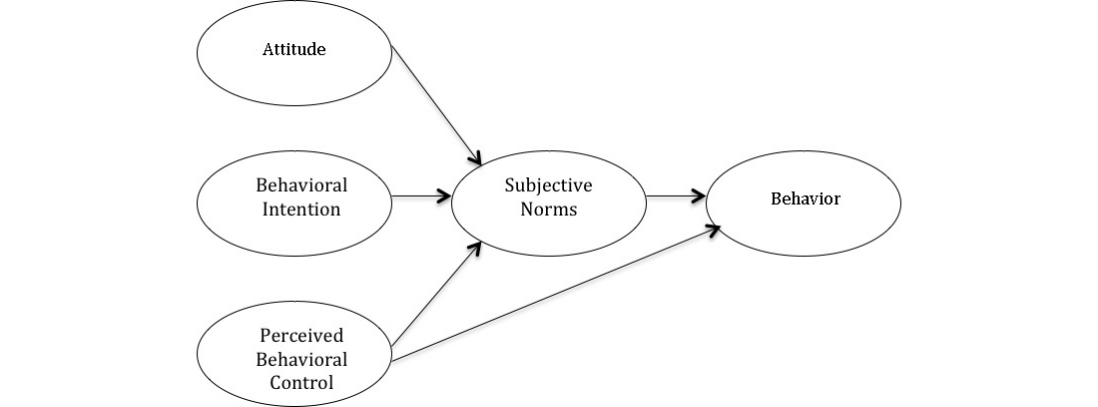
Theory of planned behavior.

The TPB provided the theoretical framework through which contributing factors and intention to use social media were explored (see Figure 1). The TPB purports that individual behavioral intention is dependent on a number of determinants that include attitude toward the behavior, subjective norms, and behavioral control relative to the behavior of interest [27,28]. Attitudes originate from an individual’s belief that the behavior, if performed, will yield an outcome they value. Subjective norms are based on normative beliefs and motivation to comply with those beliefs. For example, if important referents to the individual believe the behavior is important and the individual is motivated to follow the referent’s opinion, the subjective norm for the behavior will be positive. Perceived behavior control is belief in the ability to perform the behavior. Together, attitudes, subjective norms, and perceived behavior control are important antecedents to an intention to perform a behavior. Ultimately, behavioral intention is the most important determinant of actual behavior [29].

### Study Participants and Procedures

Participants in this study were patients aged 18 years or older of a community health center in the western United States. Community health centers were first authorized in the United States in 1975 under the Public Health Service Act. They were permanently reauthorized in 2009 in the PPACA and must be located in medically underserved areas and populations. They provide health care services that are adjusted based on ability to pay; have 51% consumer representation on their board of directors; provide medical, dental, and behavioral health care; and provide culturally competent care [26].

Following institutional review board approval from Brigham Young University, the survey questionnaire was administered using Apple iPads over a 6-week period. Each patient was invited to participate on checking in at the front desk for his or her appointment. A total of 444 patients participated in the study, the majority (302/444, 67.9%) being Hispanic followed by white (89/444, 20.2%). Qualtrics survey software was used to collect the data on Apple iPads while patients were waiting in the waiting area for appointments. A trained bilingual community health center staff member helped to administer the survey and assisted patients with any questions they had regarding the survey or use of the iPad. Every patient who checked in at the front desk of the community health center for an appointment during the study period was invited to participate. Therefore, initial contact with the patients was made in person. Patients who reported they did not have access to the Internet on a computer or cell phone were excluded from the survey.

### Measurement

This cross-sectional survey method employed the use of a 64-item closed survey questionnaire developed to assess demographics, social media use, and TPB constructs relative to social media use for obtaining health information and support. Four questions were included to collect patients’ demographic information, which included the age, race/ethnicity, gender, and annual household income of the respondent. Questions related to social media use and health were adapted from Fisher and Clayton [20] and Steele [9] (see Multimedia Appendix 1). Included in the social media response options for this study were blogs, microblogs (Twitter), social networking services (Myspace, Facebook, LinkedIn), content communities (YouTube), online group discussions, mobile phone applications, email, and texting [20]. Questions related to TPB were taken from Cameron et al [30] and modified to assess social media use in receiving health information and support (see Multimedia Appendix 1). Each construct of the TPB was assessed from 3 questions using a 5-point Likert scale with options ranging from strongly disagree to strongly agree. Cronbach alpha was used to assess the reliability of the instrument with reported reliability of .90 for attitude, .88 for subjective norm, .91 for perceived behavioral control, .88 for behavioral intention, and .86 for behavior. A Cronbach alpha of .70 and above was considered acceptable [31].

A panel of experts who reviewed the preliminary draft helped to establish content validity. In addition, the instrument was pilot tested among 50 community health center patients. Based on the feedback from experts and patients, modifications were made to several of the questions and response options. Two versions of questionnaire were created to accommodate both English and Spanish speakers. The English version was translated to Spanish, then back-translated to English to ensure that the nature of the questions were unchanged.

### Data Analysis

All statistical analyses were performed using Stata version 12.0 for Mac (StataCorp LP, College Station, TX, USA). Demographic characteristics were calculated, but nearly 23% (102/444) of demographic data were missing. Descriptive data of study variables had high response rates and statistics were calculated for each. Chi-square test statistics were computed to compare white and Hispanic respondents’ reported preferences for communicating with health care providers. Responses from nonwhite and non-Hispanic respondents were excluded from this analysis because there was not sufficient representation of other races/ethnicities to warrant this type of comparison.

Regression analyses were used to explore factors predicting behavioral intentions to use social media and actual use of social media for health purposes. Two separate models were created, one using social media use behavior as a dependent variable and a second using behavioral intentions to use social media. Using the TPB as a guide, variables were added sequentially to the respective models by block based on their conceptual proximity to the dependent variable. The model using social media use behavior included 2 blocks, whereas the model using behavioral intentions to use social media had 2 blocks. The first block in both models was comprised of demographic items, age, and gender. In the model using social media behavior as an outcome, block 2 included behavioral intentions to use social media followed by block 3, which included attitudes, subjective norms, and perceived behavioral control. In the model using behavioral intentions to use social media as an outcome, block 1 included demographics, whereas block 2 included attitudes, subjective norms, and perceived behavioral control.

## Results

A total of 444 patients participated in the study. The demographic characteristics of the respondents are shown in Table 1. More females (168/241, 69.7%) than males (73/241, 30.3%) participated with the majority being Hispanic (165/243, 67.9%) followed by white (49/243, 20.2%) and Asian (10/243, 4.1%). The majority of respondents were aged between 18 and 29 years (106/241, 44.0%) with an annual income less than US $20,000 (127/238, 53.4%).

**Table 1:** Demographic characteristics of the study participants (N=444).

Demographics		n (%)
**Gender**		
	Male	73 (30.3)
	Female	168 (69.7)
**Age (years)**		
	18-29	106 (43.9)
	30-39	64 (26.6)
	40-49	37 (15.4)
	50-59	20 (8.3)
	60-69	13 (5.4)
	≥70	1 (.4)
**Race/ethnicity**		
	African American	4 (1.7)
	American Indian	3 (1.2)
	Asian	10 (4.1)
	Hispanic	165 (67.9)
	Pacific Islander	5 (2.1)
	White	49 (20.2)
	Other	7 (2.9)
**Annual income (US$)**		
	<$20,000	127 (53.4)
	$20,000-$34,999	73 (30.7)
	$35,000-$49,999	25 (10.5)
	$50,000-$74,999	4 (1.7)
	≥$75,000	9 (3.8)
**Own computer with Internet access**		
	Yes	315 (71.0)
	No	129 (29.1)
**Own cell phone**		
	Yes	280 (92.1)
	No	24 (7.9)

### Social Media Use

Texting on a cell phone was the most common form of social media used by patients (202/274, 73.7%), followed by Facebook (152/279, 54.5%), email (123/236, 52.1%), cell phone apps (85/229, 37.1%), and YouTube (74/242, 30.6%). LinkedIn was the social media app used the least among patients (3/205, 1.5%). Compared to white patients, Hispanic patients reported more daily use of Facebook (91/155, 59%), YouTube (48/130, 36.9%), Twitter (12/108, 11.1%), online group discussions (7/106.6, 7%), LinkedIn (2/99, 2.0%), and MySpace (6.0%) (see Table 2).

**Table 2:** Daily social media use among community health center patients (N=214).

Social media type	White, n (%)	Hispanic, n (%)	*P*
Facebook	23 (46.9)	91 (58.7)	.15
MySpace	1 (2.2)	6 (55.8)	.34
LinkedIn	0 (0.0)	2 (2.0)	.33
Blogs	3 (6.5)	6 (6.2)	.94
Online group discussions	1 (2.2)	7 (6.6)	.26
Twitter	2 (4.4)	12 (11.1)	.18
YouTube	11 (22.5)	48 (36.9)	.07
Email	27 (56.3)	58 (47.2)	.29
Cell phone for texting	39 (79.6)	118 (76.1)	.62
Cell phone apps	20 (44.4)	42 (34.2)	.22

### Patient Preferences for Social Media Use in Health Care

When asked about their preference for their health care provider using social media to help them stay healthy, white respondents preferred that the provider use cell phone for texting (26/45, 57.8%), Facebook (23/45, 51.1%), and cell phone apps (20/42, 47.6%) (see Table 3). Hispanic respondents preferred cell phone for texting (89/125, 71.20%), followed by email (83/124, 66.9%) and Facebook (75/132, 57%).

With regard to sharing health information through social media, white respondents preferred email (27/45, 60.0%) and cell phone for texting (28/47, 59.6%). Hispanic respondents preferred cell phone for texting (99/137, 72.3%) followed by email (82/123, 66.7%) and Facebook (92/145, 63.5%). Analysis revealed a significant difference between white and Hispanic respondents for Facebook (16/46, 34.8% and 92/145, 63.5%, *P*<.001), Twitter (5/44, 11.4% and 31/117, 26.5%, *P*=.04), and YouTube (7/46, 15.2% and 41/116, 35.3%, *P*=.01).

**Table 3:** Number and percentage of whites and Hispanic patients who prefer that their provider use various social media to help them stay healthy and share health information (N=214).

Social media type	Help them stay healthy			Share health information		
	White, n (%)	Hispanic, n (%)	*P*	White, n (%)	Hispanic, n (%)	*P*
Facebook	23 (51.1)	75 (56.8)	.51	16 (34.8)	92 (63.5)	.001
MySpace	6 (13.6)	16 (14.7)	.87	2 (6.7)	14 (12.6)	.28
LinkedIn	3 (6.8)	12 (11.4)	.39	5 (11.1)	12 (11.4)	.96
Blogs	11 (25.0)	27 (24.6)	.95	9 (20.0)	26 (23.4)	.64
Online group discussions	14 (34.2)	22 (19.8)	.07	13 (28.9)	30 (25.6)	.68
Twitter	6 (14.6)	17 (16.4)	.80	5 (11.4)	31 (26.5)	.04
YouTube	14 (34.2)	37 (33.0)	.90	7 (15.2)	41 (35.3)	.01
Email	32 (71.1)	83 (66.9)	.61	27 (60.0)	82 (66.7)	.42
Cell phone for texting	26 (57.8)	89 (71.2)	.10	28 (59.6)	99 (72.3)	.11
Cell phone apps	20 (47.6)	47 (43.1)	.62	18 (40.9)	51 (43.9)	.73

### Factors Predicting the Use of Social Media

Mean scores of behavioral constructs revealed that Hispanics reported more intention (mean 3.07, SD 1.06) to use social media for health-related purposes than white patients did (mean 2.58, SD 0.74, *P*=.005) (see Table 4). Response options included 1=strongly disagree, 2=disagree, 3=neither disagree nor agree, 4=agree, 5=strongly agree. In addition, Hispanic respondents had a higher mean score for subjective norms (mean 3.44, SD 1.11) compared to white respondents (mean 2.92, SD 0.73, *P*=.003).

**Table 4:** Mean scores of behavioral constructs for Hispanic and white participants (N=189).

Construct	Hispanic, mean (SD)	White, mean (SD)	*P*
Behavioral intentions	3.07 (1.06)	2.58 (0.74)	.005
Attitudes	3.58 (1.29)	3.52 (0.99)	.79
Subjective norms	3.44 (1.11)	2.92 (0.73)	.003
Perceived behavioral control	3.58 (1.14)	3.66 (0.94)	.68

To explore factors that predict the use of social media within the next week for health-related purposes, a hierarchical multiple regression analysis was used in the analysis in which blocks of variables were added to the regression equation sequentially. *R* ^*2*^ refers to the overall regression equation after each block has been entered into the model; *F* for change in *R* ^*2*^ describes the contribution of each individual block (see Table 5). For block 1, the variables age and gender did not significantly account for any variance. Adding the variable behavioral intentions in block 2 accounted for 64% of the variance (*F*_3,165_=0.63, *P*<.001). For block 3, the addition of attitudes, subjective norms, and perceived behavioral control accounted for 70% of the variance (*F*_6,166_=0.06, *P*<.001). When considering all the variables entered in the model that were significant, the beta score was highest for subjective norms followed by perceived behavioral control, behavioral intention, and age.

When considering behavioral intentions to use social media for health-related purposes, hierarchical multiple regression analysis revealed that block 1 variables of age and gender did not account for any of the variance (see Table 6). The addition of attitudes, subjective norms, and perceived behavioral control variables increased the proportion of the variance to 51% (*F*_5,169_=0.51, *P*<.001). With all variables in the model, subjective norms was the only significant predictor.

**Table 5:** Predictors of social media use: contributions of each variable block to changes in *R* ^*2*^ (N=173).

Variable	Block 1 (*df*=2/170)				Block 2 (*df*=3/169)				Block 3 (*df*=6/166)			
	B	SE	*t*	*P*	B	SE	*t*	*P*	B	SE	*t*	*P*
Age	–.10	.07	–1.52	.13	–.07	.04	–1.73	.09	–.08	.04	–1.97	.05
Gender	–.10	.17	–0.58	.56	.05	.10	0.50	.61	.07	.09	0.70	.49
Behavioral intentions					.84	.05	17.46	.001	.61	.06	9.69	.001
Attitudes									–.07	.05	–1.38	.17
Subjective norms									.23	.08	3.10	.002
Perceived behavioral control									.19	.06	2.99	.003
*R* ^*2*^		.01				.64				.70		
*F* for change in *R* ^*2*^		–				.63		.001		.06		.001

**Table 6:** Predictors of intentions to use social media: contributions of each variable block to changes in *R* ^*2*^ (N=175).

Variable	Block 1 (*df*=2/172)				Block 2 (*df*=5/169)			
	*B*	SE	*t*	*P*	*B*	SE	*t*	*P*
Age	–.04	.06	–0.65	.52	–.08	.05	–1.75	.08
Gender	–.17	.16	–1.07	.29	–.16	.11	–1.39	.17
Attitudes					.11	.06	1.88	.06
Subjective norms					.59	.08	7.49	.001
Perceived behavioral control					.08	.07	1.09	.28
*R* ^*2*^		.00				.51		
*F* for change in *R* ^*2*^		–				.51		.001

## Discussion

The purpose of this study was to determine use and factors predicting use and intentions to use social media for health-related purposes among medically underserved primary care patients. The first aim of the study was to determine to what extent patients used social media. Findings indicated that social media use is common among this underserved population. The most common social media tools used were cell phones for texting (73.7%) followed by Facebook (54.5%), email (52.1%), cell phone apps (37.1%), and YouTube (30.6%). These findings are consistent with other research among non-FQHC family practice patients who reported email, cell phone for texting, Facebook, and YouTube as most commonly used sources of social media [20]. Further analysis of our findings revealed that Hispanic respondents reported greater use of 7 of 10 social media tools. These findings are similar with other research that indicates Hispanics are using social media and mobile devices at higher rates than whites [32]. The Pew Research Hispanic Trends Project reports that 68% of Latino Internet users use Facebook, Twitter, or other social networking sites compared to 58% or all Internet users in the United States [33]. A total of 86% of Latino adults own a cell phone compared to 84% of whites and 90% of blacks. Additionally, 49% of Latino adults own a smartphone compared to 46% of whites [33].

The second aim of the study was to determine patient preferences for how their health care provider should use social media to share health information and to help them stay healthy. The use of various technologies, such as emailing, texting, and smartphone apps, can enhance patient-provider relations among the underserved primary care patients [34]; however, the prevalence of health care providers’ use of various social media for communicating with patients is limited. In a survey of US doctors, 49% reported using email in the past 6 months to communicate with their patients [35]. A study in the Netherlands showed that patients’ motives for using social media for patient-provider communication were low, including only 18% for Twitter and 10% for Facebook. Similar results were found among providers; 28% said they use Twitter to communicate with patients and 14% used Facebook [12].

In general, patient preferences for their health care providers social media use are consistent with their own personal daily use of these same apps. For example, Hispanic respondents preferred their provider use cell phones for texting; using cell phones for texting was the most common social media tool used daily. For conveying health information, both groups preferred texting and email. Although the study did not specifically ask respondents what type of health information they would like to be conveyed through email or texting, personal health information may be best communicated in these ways due to privacy concerns [36]. Previous research from the Pew Internet Project has revealed that privacy concerns have led to more than half of mobile phone users uninstalling or not installing apps on their phones [37].

For sharing information to help them stay healthy, again the majority preferred texting and email, with the addition of Facebook and cell phone apps. This suggests that patients may be limited in their understanding of how providers could use a variety of social media apps to help them stay healthy. This may be because few providers have used social media for interactions with patients or that patients just have not explored that possibility. The only statistically significant differences seen between Hispanic and white respondents were related to sharing of health information using Facebook, Twitter, and YouTube, with Hispanic respondents reporting greater preferences. These differences may reflect that Hispanics use these apps more often than whites [33]. This lack of differences between the 2 groups also suggests that a social media communications strategy may not need to be based on race and ethnicity. However, a study evaluating the success of using social media to reach Hispanic cancer survivors found that this audience is very receptive to these technologies [38].

For those health care providers working to reach medically underserved community health center patients with important health information, cell phone texting and email are important to patients for health care purposes. Facebook, Twitter, and YouTube provide promising avenues of communication, especially for Hispanics. These social media applications offer an opportunity for providers to connect with underserved patients where many are interested in getting health information through social media channels. Although these avenues are promising, few studies have evaluated the use of social media for health care purposes among Hispanics [38]. Future research might explore its use in greater detail for these and other underserved populations.

A third aim of the study was to determine what factors influence the use of social media for health care-related purposes. As outlined in the TPB, attitude, subjective norms, and perceived behavioral control are important to one’s behavioral intention and behavior. Understanding the TPB factors predicting intention to use social media among patients can help to provide valuable understanding that can increase adoption of these technologies for obtaining health information. Studies have demonstrated the prominent role of social factors (ie, influence of others/groups) in predicting the use of computer technologies for health information seeking, exchange, decision making, social and emotional support, and behavior change among patients [39]. Findings from the current study revealed that subjective norms significantly predict use and intentions to use social media for health-related purposes. That is, patients in this study had higher use and intentions to use social media if important people in their lives felt the technology was important and use it for health care-related purposes. Strategies aimed at increasing the use of social media for obtaining health information and support should emphasize that important people in their life (eg, friends, family members) use social media for this purpose.

Perceived behavioral control predicted social media use but not intentions to use suggesting obtaining health information through social media channels is easier for those who are capable of using the technologies. This finding is consistent with other studies on patient use of computer technologies for health care [39] and might indicate that individuals experience barriers related to using technology to access and share health information. Barriers of this nature could include a lack of knowledge in using social media apps, costly data plans, language barriers, or health care providers that do not engage patients in such settings. Future research efforts could corroborate these findings and, if true, design strategies to minimize barriers.

Because patients are using social media as identified in this study, ignoring social media may come with risks to community health centers. These risks could come in the form of inaccurate information being shared among patients while they are online, not being aware of threats to organizational reputation, and lack of clear social media policies that can protect against liability and violation of the Health Insurance Portability and Accountability Act (HIPPA) [40]. Social media tools should be implemented by health care organizations following a planning process that includes understanding target audiences and fitting the best social media apps to meet identified communication needs [3]. Health care providers should also have clear internal and external social media use policies that guide both patient and staff involvement with the social media apps [3,41].

Although this study provides valuable insights for social media use among underserved populations, findings should be interpreted based on the following limitations. First, this study is cross-sectional and, therefore, cause cannot be assigned to any particular independent variable. Second, the study only included those individuals who reported that they had access to a computer and used the Internet. Not all patients receiving care through community health centers will benefit from a social media plan. However, many of those that are connected see value to its use for health care–related purposes. Furthermore, individuals with missing values were excluded from each analysis, which accounts for differences in the sample sizes used in each table. Missing values are not uncommon in datasets collected in locations such as FQHCs that primarily serve underprivileged individuals. Third, although reliability measures of internal consistency were acceptable, we lacked sufficient validity evidence for these scales as measures of intention to use social media for health-related purposes. Future research can help to strengthen validity evidence beyond that achieved by an expert panel. Lastly, the age range of participants included in this study included mostly individuals younger than 40 years of age. A true comparison of age would include a greater proportion of older participants. Nevertheless, we included age in multivariate analyses, but it was not significant, which may be attributable to its lack of variance. Future studies of this nature may benefit from ensuring participation from older individuals.

This study helps to demonstrate the use and factors predicting intentions to use social media among community health center patients. Community health centers deliver affordable, comprehensive, patient-centered care that is close to communities in need [42]. Optimizing primary care as motivated by the PPACA requires greater attention to advancing patient-centered medical homes, a model that community health centers value [43]. Although social media can provide another tool for primary health care providers to be even more patient-centered and provide greater personalized care [10], understanding use and factors predicting use can increase adoption and utilization of these technologies among underserved and disadvantaged patients.

## Multimedia Appendix 1

Theoretical constructs and survey questions.

## Abbreviations

FQHC: Federally Qualified Health Centers
PPACA: Patient Protection and Affordable Care Act
TPB: theory of planned behavior
