# Supplementary material for: Understanding and Predicting Social Media Use Among Community Health Center Patients: A Cross-Sectional Survey
Source: J Med Internet Res. 2014 Nov 26;16(11):e270. doi: 10.2196/jmir.3373 (PMC4260084; doi:10.2196/jmir.3373)
Supplement: Supplementary file 1 [file jmir_v16i11e270_app1.pdf]

Appendix 1. Theoretical Constructs and Survey Questions

Construct	Questions	Citation
Attitude	I think that using social media to get health information and support would:<br>· be enjoyable<br>· be useful<br>· benefit my health	(Cameron et al., 2012)
Subjective Norm	People that are important to me recommend that I use social media for getting health information and support.	(Cameron et al., 2012)
	If I use social media to get health information and support, people that are important to me would approve.	
	I think most of my acquaintances use social media for getting health information and support	
Perceived Behavioral Control	For me, using social media to get health information and/or support would be easy.	(Cameron et al., 2012)
	I feel capable enough to use social media to get health information and support.	
	I know how to use social media to get health information and support.	
Intention to Use	I plan to use social media sometime within the next week to get health information and/or support.	(Cameron et al., 2012)
	I plan to use social media in the next week to communicate with others about health.	
	I intend to use social media to post more information about my health for others to view in the next week.	
Behavior	I use social media to get health information and support.	(Cameron et al., 2012)
	I use social media to check for health updates on people that are important to me.	
	I share information about my health through social media.	
Patient Social Media Use Preferences	Social media should be used to:<br>· connect me with other patients who have the same health condition<br>· help me talk to my doctor<br>· help me get health information about my illness	(Steele, 2011)

Patient Social Media Use	How often do you use…<br>· Facebook, MySpace, Linkedin, Blogs, Twitter, YouTube, Other Online Forums, Email, Mobile phone for texting, Mobile phone apps	(Fisher & Clayton, 2012)
Preference for Health Care Provider Using Social Media	In addition to regular office visits, my health care provider should use which of the following to share health information and to help me stay healthy:<br>· Facebook, MySpace, Linkedin, Blogs, Twitter, YouTube, Other Online Forums, Email, Mobile phone for texting, Mobile phone apps	(Fisher & Clayton, 2012)
